# Hypokinetic Biliary Dyskinesia in a Pediatric Patient: A Case Report

**DOI:** 10.7759/cureus.47254

**Published:** 2023-10-18

**Authors:** Sarah Klein, Elise Quartucio, Barry Miskin

**Affiliations:** 1 Medicine, Nova Southeastern University Dr. Kiran C. Patel College of Osteopathic Medicine, Clearwater, USA; 2 General Surgery, Jupiter Medical Center, Jupiter, USA

**Keywords:** pediatric biliary disorders, pediatric, fgbd, functional gallbladder disease, biliary dyskinesia

## Abstract

The diagnosis of biliary dyskinesia (BD) in pediatric patients lacks uniformity across the literature. BD is among the most common reasons for cholecystectomy in pediatric patients. Even still, diagnostic criteria for this disorder, including symptomatology and gallbladder ejection fraction, as well as the symptomatic relief after cholecystectomy, are inconsistent across the literature for the pediatric population. We share the case of an 18-year-old female patient who presented to our clinic for cholecystectomy for a diagnosis of BD. After 10 months of seeking a diagnosis, an eventual nuclear medicine HIDA scan revealed a biliary ejection fraction of 18%, leading her to our care. The patient underwent robotic-assisted laparoscopic cholecystectomy and tolerated the procedure well, reporting a resolution of symptoms on follow-up. The diagnosis and management of BD are complicated by the combination of vague and varied symptomatology and a lack of definitive, uniform diagnostic criteria in the pediatric population. Variability in diagnostic requirements varies between sources. While some look to the interpretation of gallbladder emptying studies, others rely on surgical outcomes. To further complicate diagnosis, some patients experience symptoms that do not correlate with their gallbladder emptying studies. The controversy extends beyond diagnosis as some studies argue against cholecystectomy as a treatment for BD, as it has been shown to not always resolve symptoms. More research should be conducted to identify and establish more consistent diagnostic criteria for BD in the pediatric population, as well as to study symptomatic improvement following cholecystectomy to establish optimal treatment for these patients. Biliary dyskinesia is a relatively common but rather inconsistent diagnosis in the pediatric population, and attention should be turned toward developing uniform and consistent diagnostic criteria in order to optimally recognize, diagnose, and treat these patients, ensuring a shorter time-to-diagnosis and improved quality of life.

## Introduction

Biliary dyskinesia (BD) is a common, albeit inconsistent, diagnosis, especially in children. BD is loosely characterized by biliary abdominal pain based on location and character, a normal gallbladder present on imaging, and decreased gallbladder contraction in response to stimulus [1]. BD is part of a group of disorders described as functional gallbladder disorder (FGBD) by the Rome IV criteria to describe patients with biliary pain and an intact gallbladder without stones or sludge [2]. Diagnostic criteria for FGBD include biliary pain and the absence of gallstones or other structural pathology, and supportive diagnostic criteria include low ejection fraction on gallbladder scintigraphy as well as normal liver enzymes, conjugated bilirubin, and amylase/lipase levels [2]. The majority of BD cases are seen in adults, with the average age being 42.1 +/- 1.3, and as such, the Rome IV criteria details specific diagnostic criteria for these disorders in adults; however, no such consensus exists for the pediatric population, making the diagnosis of this disorder in children more subjective [2,3].

To further complicate the diagnosis of BD, the details of the diagnostic criteria also lack uniformity across the literature; for example, while the main diagnostic criteria in the adult population include “biliary pain,” this vagueness leads to subjective and inconsistent criteria, which can range from abdominal pain with a normal gallbladder ultrasound, to biliary-type abdominal pain, biliary colic, right upper quadrant (RUQ) and/or epigastric abdominal pain, or more vague symptoms [4]. In addition to abdominal pain, some, but not all, studies required nausea or vomiting, postprandial symptoms, fatty food intolerance, or appetite disturbance in their diagnostic criteria [4]. Furthermore, while supportive diagnostic criteria in the adult population include “low gallbladder ejection fraction (GbEF)”, the cut-off for what makes GbEF “low” is not consistent across studies [4]. The majority of the studies used a GbEF of <35%, but some utilized <40% or <50%. The method of diagnosis also varies across the literature. While most studies used cholecystokinin cholescintigraphy (CCK-CS) as their diagnostic method, some used a standard fatty meal ultrasound or Lipomul fatty meal cholescintigraphy [4]. These inconsistencies reveal the non-uniform diagnostic criteria for BD, which may complicate prompt and reliable diagnosis, especially in children.

While BD may lack a uniform diagnostic criterion in pediatrics, it is among the most common reasons for cholecystectomy in pediatric patients [5]. The most reported and well-established symptoms of BD in pediatric patients include abdominal pain (specifically the RUQ), postprandial pain, and nausea [6,7]. Between 1997 and 2010, admissions for BD in the pediatric population had a 700% increase, accounting for over 10% of cholecystectomies in children [3]. The treatment of choice for BD is well-accepted to be cholecystectomy, although there is an increasing amount of evidence that challenges this gold standard [1,8]. This evidence includes that BD is a benign disorder without risk of true complications, a majority of children experience only transient or temporary relief of symptoms postoperatively, and there are limited long-term studies that compare the benefits between operative and conservative management choices [1].

Additionally, as in our case, female patients may have more difficulty receiving an already complicated diagnosis, due to the vague symptomatology that may mimic gynecologic disorders, and therefore lengthen their time from symptom onset to diagnosis. 

The objective of this report is to highlight a case of biliary dyskinesia in a female pediatric patient in order to discuss the inconsistencies in symptomatology, diagnostic criteria, and management, and how they play a role in patient care.

## Case presentation

We share the case of an 18-year-old female patient who presented to our clinic for gallbladder cholecystectomy for biliary dyskinesia. She admitted to a 10-month-long history of progressing symptoms including nausea, shakiness or weakness, bloating, and pressure-like abdominal pain, which all occurred postprandial. She described that her pain was consistent with each meal, regardless of the food type or quantity that was consumed. She initially saw a gynecologist, whose workup yielded no significant findings, leading to a referral to gastroenterology. Amidst these appointments, the patient had an emergency department visit for abdominal pain, nausea, and vomiting for which she received a RUQ ultrasound, which revealed no acute pathology. Eventually, through her gastroenterologist, she received a nuclear medicine hepatobiliary iminodiacetic acid (NM HIDA) scan which demonstrated a biliary ejection fraction of 18% (typical normal >35%), leading her to our care.

**Figure 1:**
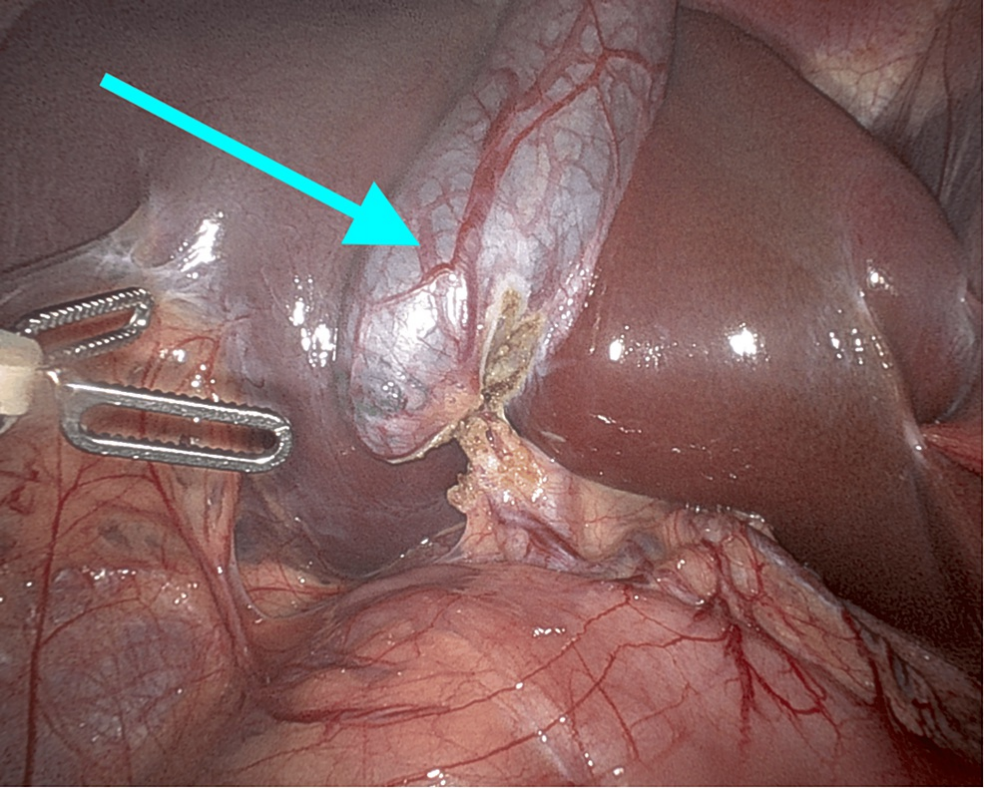
Intraoperative picture from robotic-assisted laparoscopic cholecystectomy Gallbladder (blue arrow) prior to resection

**Figure 2:**
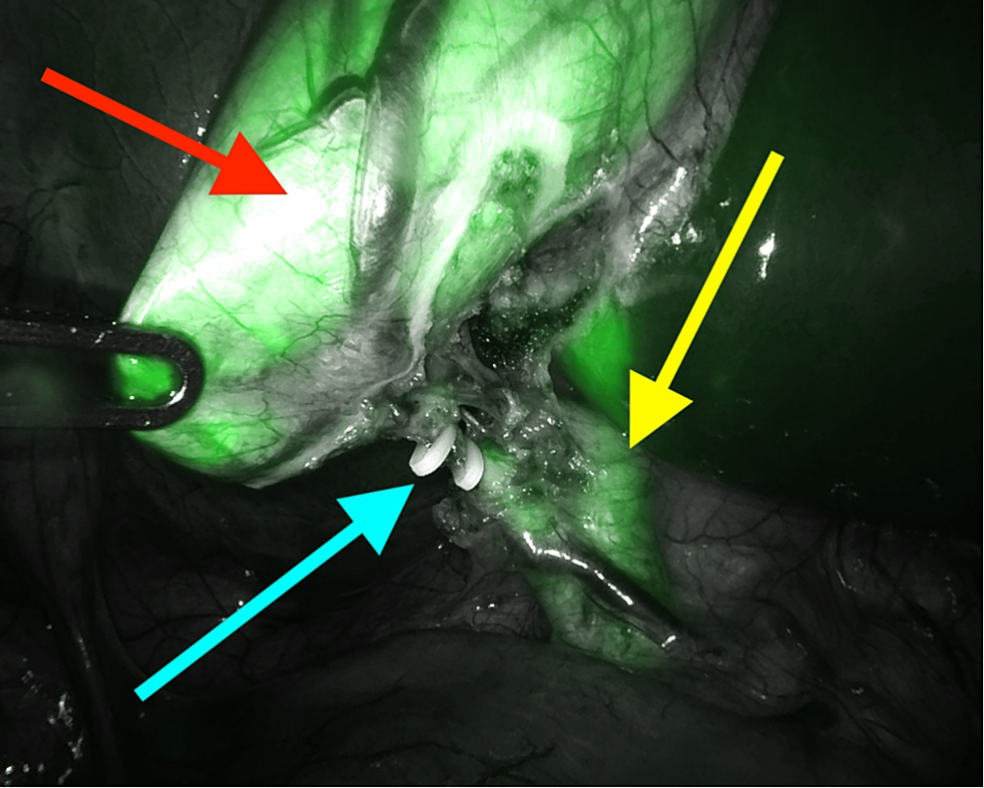
Intraoperative picture from robotic-assisted laparoscopic cholecystectomy Indocyanine green (ICG) injection fluorescence showing the clamped cystic duct (blue arrow), the common bile duct (yellow arrow), and the gallbladder (red arrow)

**Figure 3:**
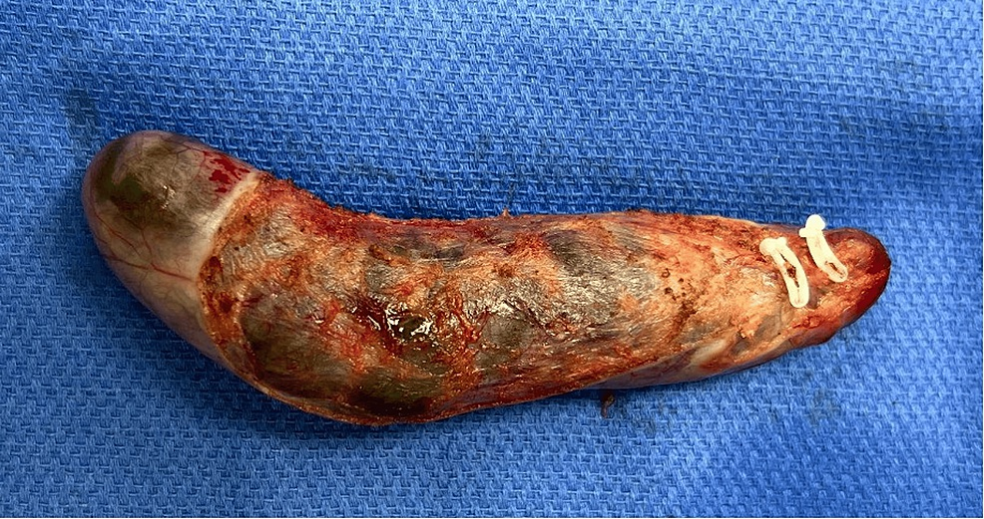
Gallbladder post-cholecystectomy

The patient was provided with treatment options and the patient elected for surgical management with a robotic-assisted laparoscopic cholecystectomy. At the time of surgery, her symptoms included abdominal pain, nausea, feeling faint, abdominal tenderness, and loss of appetite. Physical exam yielded a low-normal BMI of 18.5 and RUQ tenderness; otherwise, it was within normal limits. The patient had no significant past medical history, and no contributory family history or social history. The patient took no medications and had no known drug allergies. Preoperative vitals and labs including complete blood count and comprehensive metabolic panel were within normal limits. The patient underwent robotic-assisted laparoscopic cholecystectomy and tolerated the procedure well. The cystic duct and artery were identified and clamped, and the gallbladder was resected and removed without difficulty or complication. Intraoperative pictures can be seen in Figures 1-2. The resected gallbladder can be seen in Figure 3. On one-week postoperative follow-up, the patient was feeling well, and reported no complaints, and denied the persistence of symptoms. 

## Discussion

We chose to highlight this case of BD for a number of reasons. For a female patient of reproductive age, it stands to reason that symptoms of BD and other gastrointestinal disorders could be originally misconstrued as gynecological in origin, as they were in our patient’s case. Additionally, as mentioned previously, the diagnosis of BD is typically seen in adults and has an average age of 42.1 [3]. In our patient’s case, with her being 18 years old and on the cusp between pediatric and adult, this may complicate the diagnosis. Furthermore, we chose to highlight this case in order to bring to the discussion inconsistencies surrounding the diagnosis and treatment of BD in pediatric patients and provide support for the creation of more formal, broadly accepted diagnostic criteria as well as management options.

Outcomes following cholecystectomy in BD in children have been investigated in multiple studies and show variable conclusions. In a study by Santucci et al, investigators found that, with resolution defined as a complete cessation of symptoms without recurrence, only 66% of patients remained symptom-free at long-term follow-up (defined as six months to 11 years) [4]. Interestingly, in some studies, 52% of patients were diagnosed with a secondary condition post-cholecystectomy, such as irritable bowel syndrome, cyclic vomiting, or Crohn's disease [4]. Yet another study reported that symptoms persisted in 35.3% of patients with a median follow-up at 21 days [6]. This lack of resolution following cholecystectomy, however, is not consistent across the literature. In a study done by Liebe et al., investigators found that at two years post-cholecystectomy, 38% of patients still had resolution of abdominal pain, 43% experienced resolution of nausea, and 91% experienced resolution of their postprandial pain [7]. Similarly to those undergoing cholecystectomy for hypokinetic BD, cholecystectomy for hyperkinetic BD has not been shown to uniformly improve symptoms, with only 50% of patients in one study remaining asymptomatic at follow-up [9]. In one study by Cairo et al., investigators found that patients were undergoing significant diagnostic workup postoperatively for persistent pain, including abdominal ultrasound, esophagogastroduodenoscopy, endoscopic retrograde cholangiopancreatography (ERCP), endoscopic ultrasound, magnetic resonance cholangiopancreatography (MRCP), and colonoscopy [10]. Further, 65.2% of postoperative patients reported ongoing abdominal pain, nausea, or vomiting, and, of note, all patients who underwent postoperative ERCP with sphincterotomy reported symptom relief [10]. Secondary to these varying results across the literature, there is controversy about whether cholecystectomy should be the standard of care for BD; however, more research, including clinical trials, is indicated to support these considerations [7,11].

The management of BD is complicated by the combination of vague and varied symptomatology and a lack of definitive, uniform diagnostic criteria. Because of this, there is great variability in the diagnosis and management of functional gallbladder disorders, especially in the pediatric population [6]. The testing for BD itself comes with its own complexities. One study by Rose et al. evaluated the re-testing of GbEF in patients with BD at least six weeks after the initial test was performed. On re-testing, 53% of patients had a normal GbEF (>35%) [12]. This complicates the diagnosis of BD by undermining one of the key diagnostic tools, the hepatobiliary scintigraphy with GbEF, indicating that it is a poorly reproducible test, which weakens its use as a diagnostic tool for BD [12]. 

Additionally, there is an increasing number of studies that propose the consideration of medical management first or instead of initial surgical management for BD. Due to the oftentimes non-surgical management for other benign functional illnesses, such as dyspepsia, irritable bowel syndrome, or gastroparesis, some argue that BD should be managed similarly [13]. Although the complexities in diagnosis and treatment for BD may hinder strong randomized controlled studies from being performed, these are necessitated to provide a clearer, evidence-based management of this disorder and consider all arms of treatment [14]. 

Future research should also be conducted to evaluate the difference in the time to diagnosis from symptom onset in female versus male patients. Gender bias in medicine has been well reported in the literature; for example, it has been shown that male research continues to dominate in both animal studies and human clinical trials [15]. It has also been shown that clinical signs and symptoms, risk factors, and pharmacology can differ across genders and impact how disease is diagnosed and managed, which can impact health outcomes [15]. In fact, in a large study by Westergaard et al, investigators found that the age at first hospital diagnosis was, on average, higher in women across nearly all areas of disease [16]. Due to this, female patients may be more susceptible to prolonged symptoms, pain, and suffering due to disease than male patients may be, and thus we suggest that more research should be done in this area to evaluate whether this applies in the case of BD. We suggest that a more comprehensive review be performed in order to identify and select appropriate and accurate diagnostic criteria for pediatric patients.

The strengths of this report include that the literature shows inconsistencies in the diagnosis and management of BD in the pediatric population, so highlighting a case of a patient who struggled to arrive at a diagnosis in a timely manner and suffered as a result supports the indication that more research should be done in this area so that diagnostic and management guidelines can be strengthened for BD in the pediatric population. 

This report has potential limitations. To make our report more financially feasible, we only included articles that were freely available to the public or through the Nova Southeastern University Library. Additionally, a formal scoping or systematic review was not conducted to support this case report. As such, our report and discussion do not cover the entire body of literature on BD in the pediatric population.

Patient perspective: “I think I’m a special case in terms of diagnosis time because my mother has a lot of experience dealing with the healthcare system. She told me to write down my symptoms and to get every test the doctor thought was appropriate. She knows that it can take months until the problem is even found, much less addressed. I had a calendar full of appointments and tests, all while I was feeling awful. And even with my mom, who knew how to navigate the healthcare system like the back of her hand because of her health issues, to guide me, it took me roughly around 10 months to actually get my problem solved. I just had to keep calling and calling and hope someone would eventually help me stop feeling awful constantly.”

## Conclusions

This report highlights a case of biliary dyskinesia (BD) in a pediatric patient. This report also discusses the inconsistencies of symptomatology, diagnosis, and management of BD in the pediatric population that exist across the literature, which can largely be attributed to the lack of formal diagnostic criteria for BD in the pediatric population. This report supports more research to be done in this area in order to establish and strengthen diagnostic criteria in pediatric patients as well as diminish the inconsistencies in diagnosis and treatment prevalent within this disorder. 
